# Antecedents of immigrants’ entrepreneurial intention formation process: an empirical study of immigrant entrepreneurs in Canada

**DOI:** 10.3389/fpsyg.2023.1153142

**Published:** 2023-06-12

**Authors:** Radjabu Mayuto, Zhan Su, Muhammad Mohiuddin, Charles Fahinde

**Affiliations:** Laval University, Quebec, QC, Canada

**Keywords:** immigrant entrepreneurship, entrepreneurial intention, entrepreneurial culture distance, theory of planned behaviour, learning-based approach

## Abstract

Economic integration of ever-increasing number of immigrants in the host country is a challenge both for the immigrant and their host government. Immigrant entrepreneurship can be one of the solutions to this challenge. However, little is known about how immigrant entrepreneurship intention formation process takes place. Immigrants face various challenging situations that make them psychologically and cognitively distinct. This study models from a holistic perspective, the dimensions of individual and contextual variables as antecedents of Immigrants’ entrepreneurial intention (IEI). The study aims to identify the key factors responsible for developing EI of immigrants with an implementation intent. Cross-sectional data from Canada is examined using a sample of 250 immigrants. The analysis adopts a structural equation modelling approach. In addition to risk perception, bridging social network, and experience, we postulate that the perceived distance of entrepreneurial culture (country of origin versus host country) and entrepreneurial support are crucial factors that influence IEI. Empirical analyses based on survey data partially confirmed our hypotheses. The results show the role of psychological and cognitive factors in determining immigrants’ intention to start a new business. We extend the Theory of Planned Behaviour (TPB) by identifying certain understudied determinants in the literature and presenting a *holistic decision-making process* in the context of immigration-entrepreneurship nexus. Examining specific factors that appropriately contextualize immigrant entrepreneurship research and relativize the EI through a *learning-based approach* advances current literature. It offers insights to policymakers and practitioners to contemplate *entrepreneurial culture as a shared liability* issue (foreignness, host country), and adapt their entrepreneurship guidance accordingly. Thus, this study opens the way to a better understanding of the business behaviour of immigrants. Their impact matters for the entrepreneurial diversity that resilient ecosystems need.

## 1. Introduction

Globalization, technological advancement, and market openness have created an entrepreneurial eco-system where more and more immigrants are launching their business ventures. Challenging job market in the host country have also pushed many immigrants into entrepreneurial activities. Wage earning employment for immigrants in the current job market becomes uncertain, and they constantly seek out new opportunities to become entrepreneurs. The formation of immigrants’ entrepreneurial intention (IEI) is a process that starts with an awareness of, and initial interest in entrepreneurship, and culminates in starting that new venture (Douglas, 2020). There are both pull and push factors of IEI along with need of learning skills and knowledge to function effectively in new business eco-system. IEI is a learning process in which an individual learns the appropriate norms and behaviours to function better in the business culture of the host country. This involves an active willingness to learn and understand the host “entrepreneurial culture,” take risks to try out new, and uncertain entrepreneurial activities (Covin and Slevin, 1991).

Although over the last two decades, the study of EI in general has received great attention, studies on IEI in particular have been rather scarce (Krueger and Day, 2010; Fayolle and Liñán, 2014; Liñán and Fayolle, 2015; Mamun et al., 2017). Moreover, studies on how immigrant entrepreneurs think, and how they develop their EI and their decision to become an entrepreneur have been lacking (Douglas, 2020). Immigrants come from a different socio-economic and cultural background with various types of experiences and skills. Their thought process might be different and need an in-depth analysis to enhance our understanding as well as for policy formulation to better integrate them into the host society. This study addresses this research gap by exploring antecedents of IEI formation process which is different from EI of local entrepreneurs. There are several studies with a greater focus on understanding the factors influencing people’s intention to start a new business venture (Schlaegel and Koenig, 2014; Liñán and Fayolle, 2015). They have been explored from different perspectives, including the presence of economic and social dimensions that influence the level of Total early-stage Entrepreneurial Activity (TEA), the individual and psychological dimensions of market participants, and the processual dimension (Liñán and Fayolle, 2015; Messeghem and Torrès, 2015). While those works had contributed significantly to enhance our understanding (Douglas, 2020), the question of whether the generic determinants of EI can be generalized to all categories of individuals is still relevant (Dheer and Lenartowicz, 2018). Specifically, immigrant entrepreneurs are seen as distinct from local entrepreneurs (Wood et al., 2012), and deserve more attention from researchers and policy makers. Understanding how they think, perceive business opportunities, and make decisions to start and succeed in entrepreneurial ventures requires the development of an integrative models and consideration of specific factors that are being dispersedly addressed in current literature (Dheer and Lenartowicz, 2018).

In this study, we are exploring the factors that contribute to developing IEI from a holistic perspective. Immigrant entrepreneurship is described as the process by which an immigrant establishes a business in a country of residence which is different from their country of origin (Dalhammar, 2004). Immigrant entrepreneurship can also be an important way of integrating into the socio-economic life in the adopted country, which is also the objective of public policy in the host country. From the socio-cultural perspective, immigrants face various challenging situations that make them to be psychologically and cognitively distinct. Thus, since individuals make up their own minds “holistically” (Douglas, 2020), considering that individual factors or contextual conditions alone is inadequate in studying their entrepreneurial intention. To better understand their intentions and behaviours, it is necessary to broaden the scope of antecedents that are commonly taken into consideration in entrepreneurship research by incorporating specific factors that appropriately contextualize it. This could help to deepen the understanding of IEI construct. What are the particular factors that are relevant to the immigrant entrepreneurship context? What are the effects of the selected determinants on immigrants’ entrepreneurial intention? These are the questions that we attempt to address in this study.

An in-depth understanding of the determinants of IEI is important (Liñán and Fayolle, 2015) since immigrants and their descendants can play an important economic role through the creation of new businesses and jobs. Although many studies have identified the influence of culture on IEI models (Busenitz and Lau, 1996; Schlaegel et al., 2013; Valliere, 2019; Soltwisch et al., 2023), those studies did not explain how a culture gap impacts people’s propensity to start a new business. This is a weakness of the way culture was conceptualized in those models by an extensive use of cultural dimension grid designed to examine cultural differences between nations (Hofstede, 1980; GLOBE Project: House et al., 2004). In a migration context, exposure to different ideas and environments can affect and even transform cultural beliefs (Taras et al., 2012; Valliere, 2019). Given the increased migration and socio-professional integration problems, it becomes necessary to understand how the difference between the entrepreneurship culture of both the home country and host country influences IEI. The study intends to make following significant contributions.

First, addressing the concerns of authors such as Fayolle and Liñán (2014), Krueger and Day (2010), and Liñán and Fayolle (2015) on the paucity of IEI research, this paper extends the theory of planned behaviour (TPB) by adding more variables that are essential for immigrant entrepreneurship intention (IEI). Beyond individual factors (entrepreneurial experience, perceived risk), the cultural distance between the country of origin and host country as perceived by immigrants as well as institutional supports for entrepreneurship, have an impact on the development of the IEI. Moreover, distinct from the models of cultural value dimensions, the cognitive approach enabled us to verify that immigrants perceive cultural distance through a cognitive prism of planned behaviour (Krueger, 2003) that stimulates the feasibility of their entrepreneurship project. In addition, the existing literature lays emphasis on psychological factors such as risk perception (Alferaih, 2017; Antoncic and Antoncic, 2023). By integrating psychological and economic dimensions, our findings advance the contextualized approach in immigrant entrepreneurship and illustrate that the integrative effects of individual and contextual factors is critical for improving the quality of IEI. Therefore, we advance the research on the value of contextualizing EI and on the role of cognition in explaining this process. Secondly, we conceptualize the learning-based entrepreneurial intention following the study of Douglas (2020).

Thus, it can assist scholars in empirically studying immigrant entrepreneurship intention (IEI) in a way that is consistent with the cross-cultural understanding that reflects the reality of their research. Finally, this learning approach enables us to consider entrepreneurial culture as a shared liability issue: the liability of foreignness which requires immigrants to learn the local entrepreneurial culture and the liability of the host country which consists of finding effective mechanisms that can overcome liabilities of foreignness of newcomers to entrepreneurship.

The article proceeds as follows. In the next section, we develop its theoretical basis and the hypotheses. Then, we go further to test the hypotheses by using data from 250 participating immigrants. Thereafter, we discuss the results by highlighting the important contributions and limitations of the study before drawing a conclusion.

## 2. Theoretical foundations and hypotheses

The process of IEI development is not a linear phenomenon. Some immigrant entrepreneurs are forced to establish their own businesses as an economic survival strategy. Some others engage in ethnic enclave business and serve people from similar backgrounds and experience as them, and some other immigrant entrepreneurs belong to specific ethnic minority community that do not share the characteristics of the majority local population. Our study employs the principle of TPB and extends it by including additional predictors (Ajzen, 1991). Moreover, Ajzen states that the approach offered by the TPB provides “a conceptual framework for thinking about the determinants of the behaviour under consideration,” i.e., immigrant entrepreneurship intention, “and which can be submitted to empirical test.” However, there is scarce research attaching importance to integrating these conditions into the holistic framework of immigrants’ entrepreneurial intention formation process.

### 2.1. Theories in immigrant entrepreneurship

Early research described immigrant entrepreneurship as the process by which an immigrant establishes a business in the country of settlement different from his or her country of origin (Dalhammar, 2004). Several studies have addressed the importance of entrepreneurship on the social and economic integration of immigrants (Lofstrom, 2014; Peroni et al., 2016; Naudé et al., 2017; Duan, 2022). Immigrant entrepreneurship refers to the process whereby immigrants identify, create and exploit economic opportunities to start new ventures in their host nations” (Dheer and Lenartowicz, 2018, p.558). Conceptual understanding of immigrant entrepreneurship implies to pin down several frameworks and models that extant research provides. These theoretical views allowed researchers to explore the particularities of factors enabling or impeding immigrants to start a business venture. Rather than suggest mono-causal and then multi-causal approaches to make the bill consistent, Peroni et al. (2016) classified theories that seek to explain the relationship between immigration and entrepreneurial involvement into two broad groups: the first group focuses on the specific characteristics of immigrants for explaining differences in the propensity to start a business compared to non-immigrants; the second group relies on the institutional and cultural environment of the host country. Theories in the first group spotlight higher probabilities that make immigrants to start a new business due to (1) several forms of disadvantages in the host country (racial, linguistic, educational: *the disadvantage theory*, Wong, 1985) pushing them to necessity of self-employment instead of low paid jobs, (2) cultural traits that immigrants inherit from their home countries driving them to use ethnic resources (intra-group solidarity, financing; and shared values through ethnicity or cultural attributes, attitudes, information and advice) in areas (enclaves) where they run business belonging to the same ethnic group (*the ethnic enclave theory*: Wilson and Portes, 1980), (3) role played by certain immigrants seen as foreign traders, specifically middlemen or intermediaries between market actors (*the theory of middleman minorities*: Bonacich, 1973). Among this group, the second theory has received considerable attention through the fact of maintaining networks and connections with the country of origin.

Theories in the second group focus on the interaction between individual characteristics of immigrants and the institutions and features of the hosting societies and markets. *The interactive model* (Waldinger et al., 1990) explains immigrants’ involvement in entrepreneurship as the outcome of the interaction between their own (cultural or ethnic) resources and societies’ opportunity structures; so, immigrants mobilize their specific characteristics called ethnic strategies to access these opportunity structures. “The latter are historically shaped circumstances such as market conditions that do not require mass production or distribution, characterised by decreasing return to scale in which ethnic goods are in demand” (Peroni et al., 2016, p.642). One decade later, Kloosterman and Rath (2001) refined the former model to account for (host) country-specific institutional frameworks. Their so-called *mixed embeddedness* suggests that immigrants are not solely belonging to ethnic networks, “they are also embedded (entrenched) in specific market conditions, socio-economic and politico-institutional environments” (Peroni et al., 2016, p.642). By and large, most scholars in the field of immigrant entrepreneurship research base their arguments on the mixed embeddedness theoretical approach (Kloosterman and Rath, 2018). However, the conceptualization of this approach did not take into account the effects of countries of origin (Zhu et al., 2023). Yet “immigrants are in a unique position to create opportunities by combining and adapting ideas, products, and processes from different socio-cultural contexts” (Dheer and Lenartowicz, 2018, p.560), including but not limited to the host country. Accordingly, while scholars found immigrant entrepreneurs to be embedded in multiple contexts, literature has ignored the need to focus on the embedding process itself (Wigren-Kristoferson et al., 2022), disregarding the learning which is necessary to achieve the embeddedness (learning aspect of embedding).

### 2.2. Personal attitude, subjective norms and perceived behavioural control and immigrant entrepreneurship intention (IEI)

Entrepreneurship crystallizes the business creation process. To understand this entrepreneurial process, authors such as Bird (1988) and Krueger and Carsrud (1993) identify EI as the link between ideas and action; that, therefore, makes it so crucial in this process. For Ajzen (1991), intention captures how people exhibit their motivation and willingness to carry out the desired behaviour. Intention is one of the best predictors of planned behaviours (Armitage and Conner, 2001). However, understanding the consequences of intention requires an understanding of intention’s antecedents (Krueger, 2000).

There are various theoretical frameworks for EI (Lin et al., 2013; Schlaegel and Koenig, 2014) proposed by previous studies; each of them claims that every entrepreneurial behaviour is preceded by the intention to develop such behaviour and that several specific different factors influence this intention. The TPB (Ajzen, 1991) is probably the most applied, widely supported, and robust intention model (Krueger et al., 2000; Fayolle and Liñán, 2014; Schlaegel and Koenig, 2014; Ajzen, 2020). The theory suggests three factors that contribute to forming entrepreneurial intention: attitude towards behaviour, subjective norms, and perceived behavioural control. Attitude towards behaviour is defined as the individual’s evaluative effect towards creating a new business. The subjective norms suggest that individuals are more likely to adopt a family and close friends or mentors’ behaviours, and/or behaviours that these reference individuals/groups approve. Perceived behavioural control reflects the perceived ease or difficulty of engaging in that behaviour. Control factors include, along with many others, skills and abilities, experience, cooperation by other people, money and other resources (Ajzen, 2020) the immigrant is supposed to have acquired or learned. Several previous studies in various countries and contexts have shown the vital role played by EI (Schlaegel and Koenig, 2014; Liñán and Fayolle, 2015). Using the TPB, other studies have empirically analysed and adequately shown the positive impact each of these three antecedents has on EI (Fayolle and Liñán, 2014; Schlaegel and Koenig, 2014). According to Urban and Chantson (2019), the beleifs, values and capabilities of an individual influence the choices and decisions they make. Immigrants who believe and know that they can succeed in their entrepreneurial drive will do the needful to succeed. Immigrants with greater control of exhibiting a behaviour will have a greater intention or efforts to achieve that goal or behaviour. Attitudes, subjective norms, and perceived behavioural controls collectively form behavioural intentions, and so lead to actual behaviours (Ajzen, 1991; Mohiuddin et al., 2018). Based on the results of these studies, we formulate the following hypotheses:

*H1a*: Immigrants’ Personal attitude positively impacts their Entrepreneurial Intention.

*H1b*: Immigrants’ Subjective norms positively impact their Entrepreneurial Intention.

*H1c*: Immigrants’ Perceived behavioural control positively impacts their Entrepreneurial Intention.

**Figure 1:**
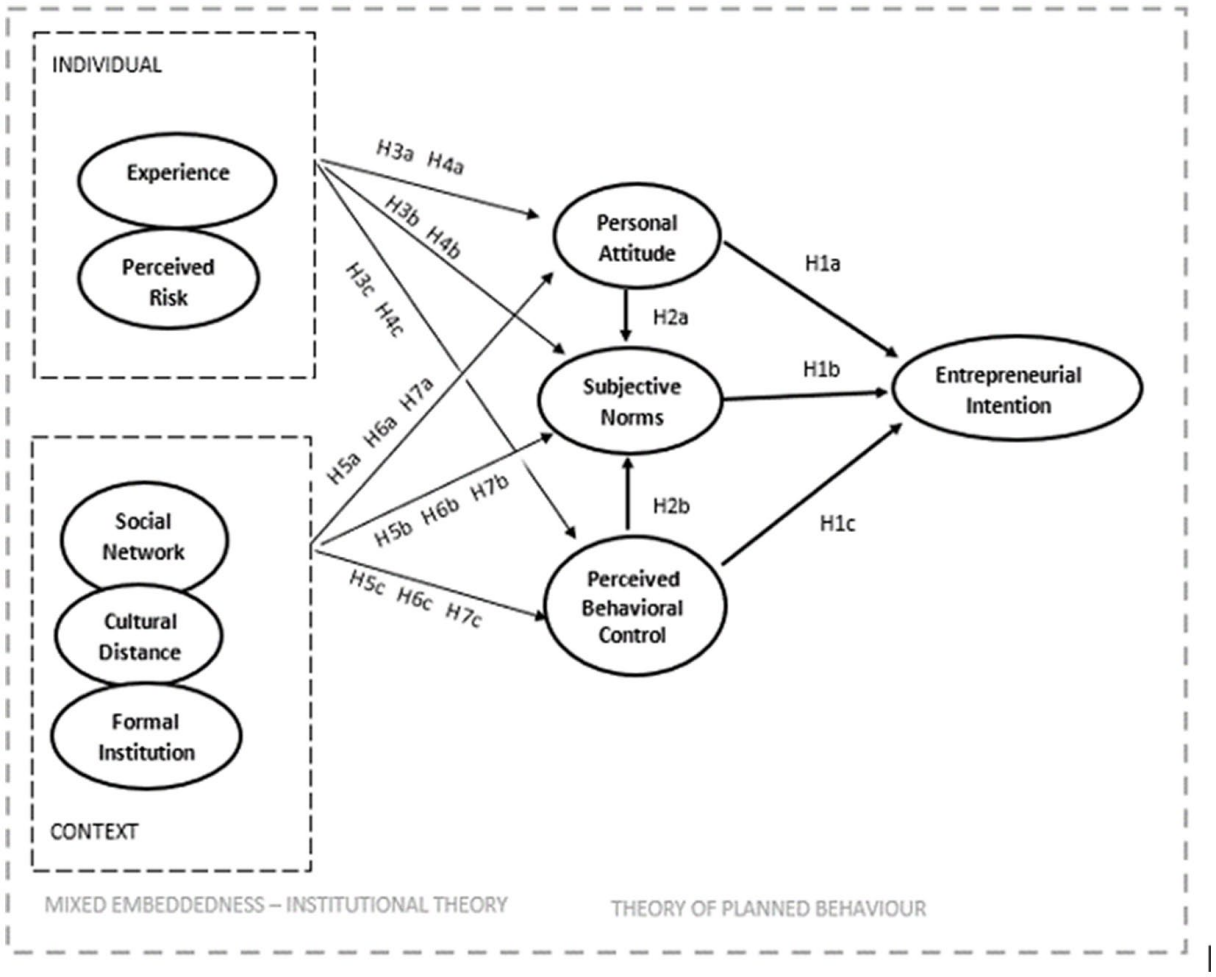
Conceptual model for predicting immigrant entrepreneurial intentions.

Our model (see Figure 1 below) aligns with the TPB on two grounds. Firstly, that there is an interaction between subjective norms and personal attitude on the one hand and behavioural control on the other hand (Ajzen, 1991). Hui-Chen et al. (2014) found a positive impact of personal attitude and perceived behavioural control on subjective norms. Still, this effect was not significant on EI. However, although the predictors of intentions are conceptually independent, empirically they are free to correlate with each other (Ajzen, 2020). Consequently, we assume an effect of personal attitude and perceived behavioural control on subjective norms. Theoretically, attitude is conceived as personal (i.e., internal) in nature and subjective norms reflect external social influence. However, attitudes have two components: personal attitude and social attitude. Behavioural outcomes can be social in addition to personal because an immigrant entrepreneur’s behaviour often has consequences for other people. Subjective norms is different from social attitude, and deals with what reference groups think about the behaviour itself. Put together, social attitude and subjective norms refer to a person’s behaviour *in relation to other people* and show the relatedness of social attitude to subjective norms. Immigrants are considered high in self-consciousness and self-monitoring, social attitudes significantly and positively influence their behavioural measures (Park, 2000). It implies that a positive attitude towards entrepreneurship of the immigrant and his own perceived feasibility of the project can contribute to the increase of perceived social pressure to engage in the entrepreneurship. This leads us to formulate the following hypothesis:

*H2a*: Immigrants’ Personal attitude positively impacts their subjective norms.

*H2b*: immigrants’ Perceived behavioural control positively affects their subjective norms.

Secondly, it aligns with the TPB in that those external factors, such as demographic (individual) or environmental (contextual) characteristics, do not directly influence intentions, but are mediated by the three antecedents of intentions (Ajzen, 1991). Furthermore, in light of Ajzen (2020), this study theorizes that the degree to which immigrants have control over the entrepreneurial behaviour will depend on their ability to overcome entrepreneurial culture distance (and perceived risk as threat or barriers of this kind) and on the presence of such facilitating factors as past experience and assistance provided by social networks and institutional context.

### 2.3. Individual variables and the antecedents of immigrant entrepreneurship intention (IEI)

#### 2.3.1. Entrepreneurial experience

Economic integration of immigrants depends on human capital which needs to be demonstrated by official qualifications (Piché and Renaud, 2018). Bureaucratic red tape and difference in international qualifications make it difficult for immigrants to assert their human capital based on foreign qualifications. To overcome this impasse, some entrepreneurs are likely to use their previous experience to carry out the entrepreneurial process, (Politis, 2005). Building on their learning, experienced entrepreneurs can identify more opportunities and generally explore more innovative opportunities with more potential for wealth creation than unexperienced entrepreneurs (Ucbasaran et al., 2010; Diacono and Baldacchino, 2022). Because experience improves their capacity to deal with liabilities of newness, effectual reasoning, and attitudes towards failure (Politis, 2008), experienced entrepreneurs must rely on it to make informed and effective decisions. Entrepreneurial experience can have a critical value in the host market. As Entrepreneurial learning transpires through, and is emergent from, practices and their relations (Thompson and Illes, 2021), prior entrepreneurial experience is expected to play a crucial role in the formation of entrepreneurial intention. This view has been empirically supported by several studies (Ucbasaran et al., 2010; Quan, 2012; Liang and Chen, 2021). Wiklund and Shepherd (2003) argue that both EI and behaviours can be conceptualized in entrepreneurs’ capabilities. Knowledge and accumulated skills of each entrepreneur are, in fact, predictors of entrepreneurial activity (Fini et al., 2009; Liang and Chen, 2021). Such is the case with experience, whether direct or indirect, which is part of human behaviour (Mueller et al., 2014) and stimulates EI. Work experience is even seen as a factor that attracts people to self-employment because it can motivate varied, well-perceived, and approved initiatives (Fisher and Lewin, 2018). The diversity of (e.g., managerial, professional) experience may benefit start-uppers, since it increases the probability of being a habitual businessperson with setting up more than one entreprise (Kurczewska and Mackiewicz, 2023). Based on the above and considering Ajzen’s (2020) view about background factors in the TPB as assumed to influence intentions and behaviour indirectly by affecting its determinants, we expect that experienced immigrants will exhibit higher personal attitude, subjective norms, and perceived behavioural control to induce the intention to start a business. Thus, we formulate the following hypothesis:

*H3*: Immigrants’ Experience positively impacts their (a) personal attitude, (b) subjective norms, and (c) perceived behavioural control towards undertaking an entrepreneurial venture.

#### 2.3.2. Perceived risk and immigrant entrepreneurship intention (IEI)

Among the personality traits considered as an antecedent of intention, the notion of risk is of particular importance (Zhao et al., 2021; Salmony and Kanbach, 2022; Antoncic and Antoncic, 2023). Risk is defined as the probability of a substantial financial loss, but the same dollar amount of possible loss could be more severe for one entrepreneur than another (Sonfield and Lussier, 2000). While risk may also involve non-financial threats like damage to reputation, it is our view that high risk seen as a threat among immigrants may arouse their own epistemic curiosity (i.e., desire for knowledge that motivates individuals to learn new ideas, eliminates information gaps, and solves intellectual problems: Heinemann et al., 2022). Generally, risk perception has been conceptualized as an assessment of risk by the decision-maker in a given situation (Mullins and Forlani, 2005). It is perceived as a determinant of risky behaviour and entrepreneurial decision-making (Fayolle et al., 2008; Antoncic and Antoncic, 2023). Despite the importance of risk on entrepreneurial intention, relatively few studies have addressed this issue (Nabi and Liñán, 2013).

Perceived risk plays an important role in the course of undertaking an entrepreneurial venture by immigrants in a new environment of their newly adopted country of residence. They must assess their abilities as well as the environment in which to create and develop the new business. The idea inherent in the evaluation process is that individuals evaluate stressful situations in terms of their well-being and especially, risk. It is important to understand to what extent perceived risk influences the formation of entrepreneurship intention. We can only assess the risk and even anticipate it when we know it, and are exposed to it, then we can have a conscious approach, called risk taking (Messikh, 2022). When forming entrepreneurial intention, risk taking allows the potential entrepreneur to temper over-optimism by determining the rate at which it will influence performance (Egele et al., 2018). Risk perception is considered as something that slows down entrepreneurial activity as it might create perception of potential losses from business activities. Thus, risk perception will negatively affect the immigrant entrepreneurial intention.

Immigrants’ willingness to take risks should be very high early in the entrepreneurial process or they would never get started. Then, this willingness may decline over time (*Ibid.*) probably when they will realize that entrepreneurial projects are exposed to many risks that require entrepreneurs to deal with and manage (Messikh, 2022). In addition to the assessment of possible damage and loss (economic, psychological and social), it is the fact of being a foreigner that creates the additional risk.

A new approach to risk in business creation argues that risk perception is context-dependent and multidimensional with two meanings: risk as a threat and risk as an opportunity (Fayolle et al., 2008). The results of a recent empirical study in a recessionary economic context indicate that both types significantly influence EI. It means that perceptions of risk as an opportunity tend to increase EI but few empirical studies have supported it. On the other hand, it is widely believed that perceptions of risk as threat tend to reduce EI (Nabi and Liñán, 2013). A similar line of reasoning can be applied to immigrant entrepreneurs who should perceive risk more as a threat than an opportunity in the host country. This can weaken the desirability and feasibility of launching such a new venture. Moreover, personality traits are background factors that are assumed to indirectly affect behavioural, normative, and control beliefs. Hence the following hypothesis:

*H4*: Immigrants’ perception of risk as a threat hurts their (a) personal attitude, (b) subjective norms, and (c) perceived behavioural control towards undertaking an entrepreneurial venture.

### 2.4. Contextual variables and the antecedents of immigrant entrepreneurship intention (IEI)

#### 2.4.1. Social network

Context is a multi-faceted concept, its social dimension reflects both the perspective of social network as well as household and family integration (Welter, 2011). Turkina and Thai (2013) define social network as a set of actors (individuals and organisations) and their links. Both “social and cultural factors may be important in the creation of entrepreneurial events and are most felt in the establishing of individual values systems” (Antoncic and Antoncic, 2023). Generally, an entrepreneur considers his entrepreneurial social network to be a medium through which he may gain access to different resources. Links in social networks are often divided into two types: (1) close or strong ties are intimate bonds that exist between an (immigrant) entrepreneur and family members, very close friends, or other members of ethnic group, and (2) loose or weak ties form social ties that are more diffuse, less intense, and often short-lived than is true for close or strong ties (Baron and Hmieleski, 2018). Bonding social networks emphasizes on the density and tightness of the existing social network of an individual, while bridging social networks concentrate on an individual’s scope and type of social network ties and their impact on the several outcomes including venture creation and nascent behaviour.

Literature on immigrant entrepreneurship insists on the propensity of entrepreneurs to rely on ethnic networks to mobilize resources that are useful for the creation of their business (Dheer and Lenartowicz, 2018; Webster and Kontkanen, 2021; Zhu et al., 2023). This understanding enlightens Granovetter’s (1985) use of the social embeddedness concept to measure the effects of social networks on economic behaviour. The cultural anchoring of immigrants would facilitate the mobilization of ethnic resources as well as a vertical integration with co-ethnic producers, resellers, and customers (Dheer and Lenartowicz, 2018). This vertical social embeddedness provides physical and emotional support through internal interactions and promotes trust among internal ethnic group members by sharing information. Beyond the kinship-based connections in the host country, high-quality social relationships are supposed to be built and nurtured with purpose. What we think of as horizontal social embeddedness facilitates entrepreneurs to obtain accurate information from outside the community and identify opportunities in the wider society, as well as gain decision-making advantages. For this purpose, healthy entrepreneurial bridging social networks should provide contacts and relationships to improve entrepreneurial capability building (Zhao et al., 2021). Nowadays, following the sobriety of ethnic enclaves (Ndofor and Priem, 2011) and the boom phenomenon of entrepreneurial digital social networks, this can allow immigrants to update information about new markets and opportunities, leading them to a more adequate decision (Dileo and Pereiro, 2019; Zhao et al., 2021). Ultimately, what is important is the strong entrepreneurial awareness to which the interaction of the role of potential entrepreneurs (embedded in social networks) and the social context leads (Zhu et al., 2023). Moreover, since “Mobilities are applied to immigrant entrepreneurship to investigate the ways immigrants build links and bridges through spaces and places” (Webster and Kontkanen, 2021, p.223), such horizontal social embeddedness is beneficial for immigrants particularly in establishing reputation, improving performance (Liang and Chen, 2021; Zhang et al., 2022) and enhancing legitimacy. Different levels of networks can promote the circulation and exchange of resources (Wang et al., 2021).

Research evidence suggests “a statistically significant relationship between (1) bonding social capital and perceived desirability, (2) bridging social capital and perceived feasibility, and (3) entrepreneurial intentions and both perceived desirability and perceived feasibility” (Abebe, 2012). Current research on bridging social capital derived from entrepreneurs’ social networks indicates that it indirectly influenced EI through the positive mediation of entrepreneurial self-efficacy, with strong significant relationships between it and entrepreneurial self-efficacy (Liang and Chen, 2021; Ferreira et al., 2022). Following the above literature and drawing on the TPB, apart from the internal co-ethnic (bonding) network, we postulate that external (bridging) network, which goes beyond ethnicity, will have positive impact on personal attitude, subjective norms and perceived behavioural control translating into higher level of IEI. Those who develop diverse, outwardly oriented (inter-ethnic) networks will be more likely to show higher intentions than their counterparts who have less or no such network within the social system. The following hypothesis is then formulated:

*H5*: Immigrants’ ability to bridge social network positively impacts their (a) personal attitude, (b) subjective norms and (c) perceived behavioural control to undertake an entrepreneurial venture.

#### 2.4.2. Cultural difference

Institutional context which draws on the concept of formal and informal institutions, as coined by North (1990) in terms of “the rules of the game in a society,” are humanly designed systems that shape human interaction. When it comes to informal institutions (norms and attitudes in a society), the strand of literature studies its influence on relationship to entrepreneurship. Such research draws attention to the impact of culture (Welter, 2011). Culture has been described as “the interactive set of common characteristics that influence the response of a human group to its environment” (Hofstede, 1980, p.21). International entrepreneurship scholars argue that a country’s values, beliefs, and norms affect its residents’ entrepreneurial orientation (Busenitz and Lau, 1996). Therefore, entrepreneurs’ effectual behaviour differs due to their national cultural traits (Strauß et al., 2020). Regarding entrepreneurial culture, it is seen as one that values the personal characteristics associated with entrepreneurship (Maldonado et al., 2021). Hence, conceptualization, manifestations, and consequences of entrepreneurial culture are inherently central to entrepreneurial intention research. From a microlevel standpoint, this concept refers to an organizational culture embodying and upholding entrepreneurial attributes and characteristics (Genoveva, 2019; Hassan et al., 2021). From a macrolevel viewpoint, entrepreneurial culture is found as a component of national culture that enables the success of economic growth (Valliere, 2019). Accordingly, GEM Reports on Canada experts’ opinion claim that cultural environment is one of the most favorable conditions for entrepreneurship in the country (Langford et al., 2015; Gregson and Saunders, 2019). Many developed and developing economies have allocated and spent vast amounts to promote and cultivate entrepreneurial culture, a valuable entrepreneurial ecosystem dimension (GEM, 2020). For institutions to foster the entrepreneurial spirit among people seeking to enter business, it becomes important to do so by focusing on empowering the individuals to carry out their entrepreneurial tasks (Hassan et al., 2021). We consider entrepreneurial culture as an important factor in forming and promoting immigrants’ entrepreneurial behaviour and their integration in the entrepreneurial ecosystem of the host country. Thus, immigrant’s entrepreneurial acculturation should be placed in a lifelong perspective and started before business creation.

Moreover, if foreignness is inherent to mobility, distinguishing between entrepreneurial cultures is central to immigrant entrepreneurship where culture is seen to be a liability issue. As with the liability of foreignness, which depends mainly on the social, relational and institutional factors, the difference in entrepreneurial cultures of the home and host countries can give rise to unfamiliarity and lack of embeddedness, discrimination by host country actors, and relational hazards for immigrants in their host countries (Eden and Miller, 2004). Lack of social legitimacy associated with information asymmetry and economic nationalism in host countries can give rise to a lack of trust in immigrants’ entrepreneurial ventures, and sometimes even discrimination against them (Kim, 2019). On the other hand, in a new country, immigrants might have certain emotions vis-à-vis this environment. Hofstede et al. (2004) suggest that the tension created by the differences between the cultural dimensions and the country’s institutions can also be a source of dissatisfaction (Schlaegel et al., 2013). Generally, this perspective suggests that dissatisfaction among individuals who do not conform to the predominant cultural dimensions at the country level would induce the tendency to start their own business.

Dissatisfaction could arise when there is a cultural clash between the immigrant and the informal environment of his host society. Missing the cultural embeddedness boat, some immigrants may feel that they are not affected by some of the values and practices that underpin the social fabric of their host country. Overall, this distancing could somehow influence the entrepreneurial decision in the same theoretical and empirical logic as in the recent studies results (Porfírio et al., 2023). The former confirmed the importance of entrepreneurial culture and education to promote entrepreneurial self-efficacy and then develop EI. The latter revealed entrepreneurial culture has a positive influence on attitude. Based on the foregoing, we assume that the perceived culture distance will decrease the IEI levels by lowering the effects of personal attitudes, subjective norms and perceived behavioural control. Instead of leveraging EI, it will hold a negative impact on both attitude, subjective norms, and perceived control. The following hypothesis is therefore formulated:

*H6*: Immigrants’ perceptions of Distance in entrepreneurial culture lower their (a) personal attitude, (b) subjective norms, and (c) perceived behavioural control to undertake an entrepreneurial venture.

#### 2.4.3. Formal institutions

When it comes to formal institutions, the critical role that institutional context plays in venture creation process has been well-documented in the wider entrepreneurship literature (Lyons and Zhang, 2017; GEM, 2020; Kim and Lee, 2020; Valencia-Arias et al., 2022; Zhang et al., 2022). Formal institutions as political and economy-related rules which create or restrict opportunity areas for entrepreneurship (Welter, 2011) provide the framework and structure to facilitate some types of exchange and the framework in which people have confidence in determining outcomes (North, 1989). They reduce uncertainty by providing a stable platform for human interaction (Salimath and Cullen, 2010), and affect the costs of interactions in such an environment by providing incentives for some behaviours and discouragement for others (Audretsch and Keilbach, 2004). The availability and quality of institutional context that produce human capital through education, training, and/or learning-by-doing should be likely to generate a strong propensity for new businesses. Therefore, in this study, we argue that institutional support for entrepreneurship has established itself as a critical policy direction to address the structural issue of the lack of employment opportunities, particularly in a hostile or corrosive environment of immigrants.

The literature conceptualizes formal institutions in terms of the financial and educational support attributed or attributable to entrepreneurship (De Clercq et al., 2013) and related regulations and the perception of corruption (Bowen and De Clercq, 2008). These factors that motivate entrepreneurship are generally evoked from either a positive angle (effectiveness, efficiency, etc.) or a negative angle (lack, poor adequacy, etc.). According to Zhang et al. (2022), government support falls into two categories: (1) to give policy support (i.e., tax reduction, exemption, partial discount loan) and (2) to provide service support for solving problems (i.e., simplifying procedures of entrepreneurial qualification, broadening the channels of entrepreneurial credit loans, providing entrepreneurial technical guidance and training, and building a business interaction platform that is conducive to entrepreneurship). In this vein, entrepreneurship training program as examined by Lyons and Zhang (2017) is informative. The authors found that the effect of such program was small for minorities in the short run, more pronounced for minorities’ likelihood of longer run start-up activity, whereas for non-minorities the effect was small and statistically insignificant. Entrepreneurial supports have a positive influence on entrepreneurial intention (Abebe, 2012; Im, 2013; Kim and Lee, 2020). The more an immigrant perceives an institutional support and the more training he receives, the more entrepreneurial attitude he will have (Valencia-Arias et al., 2022). Thus, drawing on the TPB, we expect institutional support for immigrant entrepreneurship will positively influence both attitude, subjective norms and perceived control of the immigrants for their entrepreneurial career intention.

Based on the above, it can be inferred that immigrant entrepreneurs who have favorable perceptions of formal institutional environment and supports for entrepreneurship will have higher EI. The following hypothesis is then formulated:

*H7*: Immigrants’ perceptions of Entrepreneurial supports (institutional) have a positive impact on their (a) personal attitude, (b) subjective norms, and (c) perceived behavioural control towards undertaking an entrepreneurial venture.

## 3. Methodology

### 3.1. Empirical setting

This study focuses on immigrants living in Canada regardless of their countries of origin. Recently, innovations in the GEM (2020) methodology have made it possible to assess the quality of an economy’s entrepreneurial ecosystem or the entrepreneurship environment, graded over 10 points. According to the 2019 NECI (National Entrepreneurship Context Index), out of the 5 participating economies that we consider as traditional countries of immigration (TCI), Canada is the second country with the most favorable environment for entrepreneurship after the United States of America. Moreover, in its 2019 Budget, the Government of Canada extended its support for inclusive entrepreneurship (for youth, women, seniors and indigenous people). With the proven success of economic-class immigrants in entrepreneurial activities, it is clear that this economy has an environment that is the most conducive (for an explanation of why this matters, see Guerrero et al., 2020). This is the reason why we decided to base our work on immigrants in Canada. The following procedure and instruments were known to and approved by the ethics committee of the Laval University.

### 3.2. Sample and procedure

Our data draw from a survey conducted from June 2018 to July 2019 through the following sampling procedure. We were able to contact the immigrants through MIFI (*Ministère de l’Immigration, de la Francisation et de l’Intégration*), where we accessed the TCRI (*Table de concertation des organismes au service des personnes réfugiées et immigrantes*) to obtain a list of immigrant associations registered in their database. The list enabled us to contact the presidents of several African, European, Latin-Caribbean, and Asian immigrant associations. With the help of the associations’ presidents, we were able to send our questionnaire to a total of 444 participants. The questionnaire was developed in English and French, the two official languages of Canada. To administer it to the participants, we combined two data collection techniques, namely an online survey via an electronic link and manual completion of printed copies of the questionnaire. For the online survey, an email with the electronic link to the survey was sent to association presidents, who then forwarded it to their respective members. Printed copies of the questionnaire were distributed to participants during association gatherings such as community fairs, general meetings, and open events. The questionnaire items were formulated in such a way as to minimize social desirability bias. Also, respondents were assured of the anonymity of their responses (Kautonen et al., 2015). Out of the 444 distributed questionnaires, 275 were returned by respondents. Out of these 275 returned questionnaires, we had to reject 25 based on incomplete answers. Therefore, 250 valid questionnaires were finally retained. This sample of 250 respondents was made up of 54% Africans, 26% Europeans, 10% Latin-Caribbeans, and 10% Asians. From a gender perspective, the sample was made up of 27% men and 73% women. As for the sectors of activity envisaged for their future businesses, among the 101 participants who answered this question, 18% targeted restaurants, 15% groceries, 12% services related to new technologies, and 9% retail sales (see Table 1).

**Table 1:** Demographic breakdown of participants.

Region of origin	Women	Men	Total		Sectors (available data, number of cases)
Africa	107	28	135	54%	Restaurant (15); grocery (7); retail industry (6); computer-related service (7); driving school (2), truck transportation (2)
Asia	23	1	24	10%	Agriculture contractor (5); convenience store (5); garage (1); restaurant (1); perfumery (1)
Europe	37	29	66	26%	Computer-related service (4); immigration counselling (2); retail industry (1); construction (1); grocery (1)
Latin America & Caribbean	16	9	25	10%	Computer-related service (2); agri-food industry (2); fruits-vegetable import (1); restaurant (1)
Total	183	67	250		Act
	73%	27%			

### 3.3. Measurement of the variables

#### 3.3.1. Measurement of EI and its antecedents

*Entrepreneurial intention* and its antecedents were measured using the items developed by Liñán and Chen (2009) on a 7-point Likert scale ranging from 1 (strongly disagree) to 7 (strongly agree). Thus, a set of 4 items, including statements such as “I am ready to do anything to be an entrepreneur” and “My professional goal is to become an entrepreneur,” were used to measure respondents’ EI. An analysis of this construct’s reliability shows that it has good internal consistency with a Cronbach’s alpha of 0.77. The immigrants’ *attitude towards entrepreneurship* was measured using four items indicating their attractiveness and the level of benefits they perceive for entrepreneurship. These items include statements such as “Being an entrepreneur would give me great satisfaction” and “A career as an entrepreneur is attractive to me.” This construct has a Cronbach’s alpha of 0.68. To measure *subjective norms*, we used four items including statements such as “My immediate family would approve of my decision to start a business” and “My friends would approve of my decision to start a business.” Cronbach’s alpha associated with this construct is 0.75. *Perceived behavioural control* was measured with three items, including statements such as “If I tried to start a firm, I would have a high probability of succeeding” and “I know how to develop an entrepreneurial project” with a Cronbach’s alpha value of 0.72.

#### 3.3.2. Measurement of individual variables

Two variables – risk perception and entrepreneurial experience – were used to analyze individual factors’ effect on immigrants’ EI. We adapted the items developed by Nabi and Liñán (2013) to measure the perception of risk as a threat on a 7-point Likert scale ranging from 1 (strongly disagree) to 7 (strongly agree). These items include statements such as “Starting a new business is very risky” and “The probability of a new venture doing poorly is very high,” giving us a Cronbach’s alpha of 0.68. To measure *Entrepreneurial experience*, we asked respondents to indicate the number of years they have already spent doing business in Canada.

#### 3.3.3. Measurement of contextual variables

To analyze the effect of contextual factors on immigrants’ EI, we used three variables: Social network, Distance of entrepreneurial culture, and Institutional context. *Social network* was measured using three items adapted from Lancee (2010) on a 7-point Likert scale ranging from 1 (strongly disagree) to 7 (strongly agree). Lancee (2010) identifies two types of networks: Bonding network and bridging network. However, in the present study, bonding network items do not have an excellent internal coherence. We, therefore, had to select only *bridging network*, which enables us to assess the extent of immigrants’ weak social ties with the host society. The items used include statements such as “I establish relationship more with native Canadians than with my ethnic group,” and the construct’s internal reliability test shows a Cronbach’s alpha of 0.69.

We used four items from St-Jean and Duhamel (2015) in terms of distance of entrepreneurial culture. They postulate that entrepreneurial culture can be perceived through the media coverage given to entrepreneurship and entrepreneurs’ social status. The other two items are from Langford et al. (2015), for whom a society’s entrepreneurial culture can be perceived through the entrepreneurial spirit and propensity to risk that characterizes individuals and their creativity and ability to innovate. Since our focus in this work is the difference in entrepreneurial culture between Canada and the immigrants’ countries of origin, we adapted the items developed by St-Jean and Duhamel (2015) and those of Langford et al. (2015) in the same manner as Saha et al. (2011) who studied the role of cultural distance in the medical field. Thus, we asked respondents to indicate the magnitude of the difference they perceive between Canada’s entrepreneurial culture and that of their countries of origin on a 7-point Likert scale ranging from 1 (very similar) to 7 (very different). Items used include statements such as “Media attention towards entrepreneurship in Canada and my country is…” and “The way status is accorded to successful entrepreneurs in Canada and my country is…,” with a Cronbach’s alpha of 0.88.

*Formal institution* variable was measured by asking respondents to indicate their perception of entrepreneurs’ assistance by formal institutions (Turker and Selcuk, 2009). A set of five items was used to measure this variable on a 7-point Likert scale ranging from 1 (strongly disagree) to 7 (strongly agree). These items relate to several aspects of institutional support, such as access to funding (Albert et al., 2003), support for innovation (Schwarz et al., 2009), networking arrangement (Bakkali et al., 2010), etc. For example, the items used include statements such as “In Canada, training to be an entrepreneur makes funding available for business start-up” and “In Canada, support structures enable joining or developing social networks.” For this construct, the internal reliability test shows a Cronbach’s alpha of 0.74.

## 4. Results

### 4.1. Validating the measurements

We tested the different scales used to ensure their convergent validity, discriminant validity, and reliability. To this end, an exploratory factorial analysis was carried out on all the items making up each of the multi-item variables used in our IEI model. We first verified the relevance of the factorial analysis by using the Kaiser-Meyer-Olkin (KMO) test to evaluate the homogeneity of the items. The results presented in Table 2 below show that there is a factorial solution for each of the variables because the KMO values are more significant than 0.5 (Kaiser, 1974). In addition, the factor analysis confirmed the unidimensional structure of the scales used because, for each of the variables, only one eigenvalue is greater than 1. The proportion of the total variance explained by each factor is also consistent with the recommendations in the literature (Child, 1990). In addition to the exploratory factorial analysis, we used Cronbach’s alpha to examine the internal reliability of each of the constructs used in our model. The results of this test reveal that the different constructs have an acceptable level of internal consistency.

**Table 2:** Validity of the measurements.

Constructs and variables	Cronbach’s alpha	KMO	Eigenvalue	Cumulative variance %	Factor loading
Entrepreneurial intention	0.773	0.701	2.397	59.918	
Int1					0.647
Int2					0.857
Int3					0.734
Int4					0.840
Personal attitude	0.681	0.731	2.083	52.087	
Att2					0.645
Att3					0.796
Att4					0.698
Att5					0.740
Subjective norms	0.751	0.75	2.298	57.462	
Norm1					0.738
Norm2					0.733
Norm3					0.822
Norm4					0.736
Perceived behavioural control	0.726	0.645	1.939	64.629	
Ctl1					0.798
Ctl3					0.861
Ctl4					0.750
Perceived risk	0.683	0.681	2.085	52.129	
Risk1					0.633
Risk 4					0.652
Risk6					0.767
Risk8					0.819
Cultural distance	0.881	0.833	2.972	74.300	
Cul1					0.891
Cul2					0.886
Cul3					0.882
Cul4					0.784
Formal institution	0.735	0.714	2.457	50.142	
Inst1					0.601
Inst2					0.667
Inst3					0.658
Inst5					0,716
Inst6					0.840
Bridging social network	0.693	0.575	1.875	62.490	
Bsn4					0.525
Bsn5					0.570
Bsn6					0.780

In addition, the mean, standard deviations, and bivariate correlations between the independent and dependent variables are presented in Table 3. In this study, correlations analysis confirmed a statistically significant relationship between EI and all its three antecedents, according to the TPB. There is also a statistically significant relationship between some exogenous variables and EI antecedents (endogenous variables). Testing further for structural validity (Fornell and Larcker, 1981), the square root of the variance shared between the constructs and their measures (average variance extracted, AVE) was analysed. Table 3 reveals that the square root of AVE is in all cases larger than the bivariate correlations, thus confirming structural validity.

**Table 3:** Correlation matrix and descriptive statistics.

	Mean	SD	AVE	Intention	Attitude	Norms	Control	Cultural	B. network	Institution	Risk
Intention	6.131	0.7018	0.599	1**(0.774)**							
Attitude	6.483	0.4841	0.521	0.348^***^	1**(0.722)**						
Norms	6.37	0.5075	0.575	0.329^***^	0.225^***^	1**(0.758)**					
Control	5.5893	0.6211	0.647	0.112*	0.177^***^	0.1	1**(0.804)**				
Cultural D.	5.558	1.0179	0.920	−0.031	−0.049	−0.053	−0.189^***^	1**(0.959)**			
BS network	3.7983	0.9020	0.403	0.043	0.113*	−0.059	0.08	−0.167^***^	1**(0.635)**		
Institution	5.602	0.6282	0.491	−0.037	−0.054	0.061	0.075	−0.059	0.071	1**(0.701)**	
Risk	4.543	0.6806	0.811	0.04	−0.126^**^	0.129^**^	0.154^**^	−0.194^***^	−0.062	−0.135^**^	1**(0.901)**

(*) = Significant at 10%; (**) = Significant at 5%; (***) = Significant at 1%; *N* = 250; SD = Standard deviation; AVE = Average Variance Extracted. Bold diagonal elements in brackets represent the squared root of the AVE.

### 4.2. Path model’s test

It should be stated from the outset that we tested two models in succession, one with a social network (Model 1) and the other without a social network (Model 2). Since social network ties (H5a,b,c) were all found to be insignificant, this variable and all other insignificant relations (H3a,b; H4c; H6a,b; H7a,b) were removed from the initial model to test whether the adjustment would, at best, improve or, at worst, be maintained.

The results presented in Table 4 below show that the structural model proposed without considering the network (Model 2) is overall well fitted and can therefore be used to test our different research hypotheses. All the models were tested with MPlus 6.1 (Muthén and Muthén, 2010). The criteria used to assess the fit quality of this model are the Chi-square ratio to the degree of freedom (χ2/df = 1.401), the Root Mean Square Error of Approximation (RMSEA) = 0.040, the Comparative Fit Index (CFI) = 0.968, and the Tucker-Lewis Index (TLI) = 0.923. A comparison of these different indicators’ values with those recommended in the literature: χ2/df ≤ 3, RMSEA ≤0.1, CFI ≥ 0.90, TLI ≥ 0.90 (Segars and Grover, 1993; Gefen et al., 2000) shows that the estimated model is well fitted. Moreover, Chi-square is insignificant. In addition, most of the estimated relationships are significant. Except for H1c and H2b, all the other research hypotheses are validated. Thus, our results show that immigrants’ attitudes toward entrepreneurship and their perception of subjective norms positively affect their EI. Hypotheses 1a and 1b are therefore validated. The results also indicate that H3c, H4a, and H4b on the relationships between individual variables and the antecedents of intention are validated. Similarly, the hypotheses on the relationships between contextual variables and perceived behavioural control (H6c, H7c) are validated. As for the relationships between EI antecedents, Hypothesis 2a, which postulates that attitudes towards entrepreneurship positively affect subjective norms, has been validated.

**Table 4:** Results of structural model.

Hypothesis	Hypothesized Path	Hypothesis	Hypothesis	Hypothesis
H1a	Personal attitude → Intention	0.284***	0.283***	Support
H1b	Subjective norms → Intention	0.263***	0.262***	Support
H1c	Perceived behavioural control → Intention	0.035	0.035	No support
H2a	Personal attitude → Subjective norms	0.246***	0.238***	Support
H2b	Perceived behavioural control → Subjective norms	0.040	0.034	No support
H3c	Experience → Perceived behavioural control	0.405***	0.406***	Support
H4a	Perceived risk → Personal attitude	−0.119**	−0.126**	Support
H4b	Perceived risk → Subjective norms	0.149**	0.154**	Support
H6	Cultural distance → Perceived behavioural control	−0.173***	−0.179***	Support
H7	Formal institution → Perceived behavioural control	0.123*	0.126*	Support
H5a	Bridging social network → Personal attitude	0.105		No support
H5b	Bridging social network → Subjective norms	−0.081		No support
H5c	Bridging social network → Perc. behavioural. Control	0.039		No support
Model (1)	*χ*2/df = 1.296 (p = 0.206); RMSEA = 0.034; CFI = 0.967; TLI = 0.924			
Model (2)	*χ*^2^/df = 1.401 (p = 0.156); RMSEA = 0.040; CFI = 0.968; TLI = 0.923			

S. Estimates = Standardised values, *** = Significant at 1%; ** = Significant at 5%; * = Significant at 10%.

The results of Model 1 are also presented in Table 3; they show that the structural model incorporating social networks is generally well-fitted, as was the previous model. The adjustment indices show that the model fits well with the data: the Chi-square ratio to the degree of freedom (*χ*^2^/df) = 1.296, the Root Mean Square Error of Approximation (RMSEA) = 0.034, the Comparative Fit Index (CFI) = 0.967, and the Tucker-Lewis Index (TLI) = 0.934. Apart from the hypotheses about bridging social networks, which appear to have no significant effect, all relationships in the model maintained their significance level.

**Figure 2:**
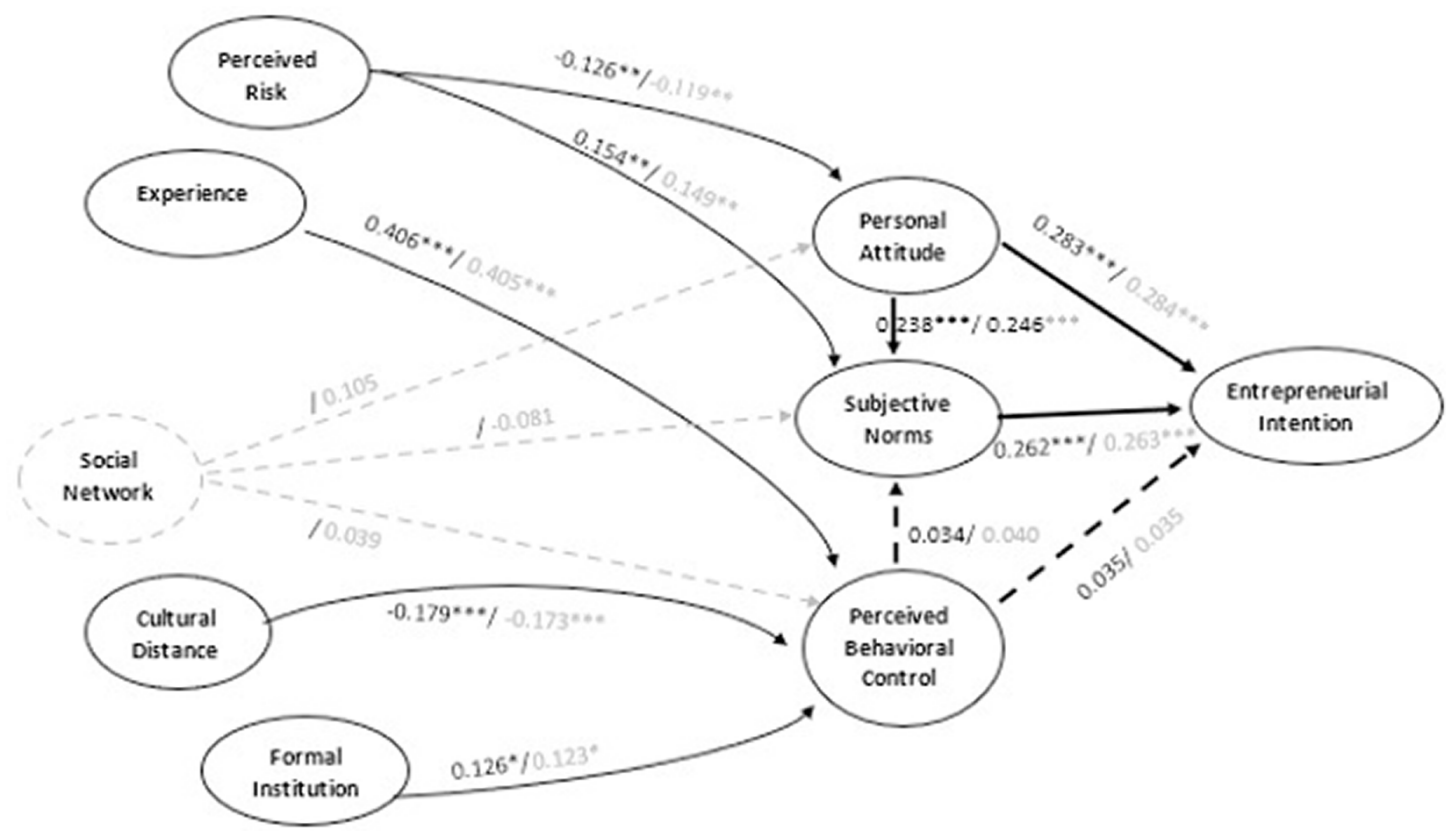
Final empirical model for predicting immigrant entrepreneurial intentions.

Figure 2 summarizes the results of the final empirical model with all the relationships tested. When comparing the standardised coefficients represented in this Figure, it is clear that the magnitudes of the standardised coefficients differ. Perceived risk as a threat was related to personal attitude (β = −0.126, significant at 5%) and subjective norms (β = 0.154, significant at 5%), confirming H4a and H4b, respectively. Entrepreneurial experience was related to perceived behavioural control (β = 0.406, significant at 1%), which partially confirms H3. Cultural difference in entrepreneurship was related to perceived behavioural control (β = −0.179, significant at 1%), which partially confirms H6. Institutional entrepreneurial support offered by formal institution was linked to perceived behavioural control (β = 0.126, significant at 10%), which partly confirms H7.

## 5. Discussion on findings of this study

In this article, we studied the antecedents of immigrants’ entrepreneurial intention (IEI) by testing a model that combines previous research on entrepreneurship, immigrant entrepreneurship, and psychological factors that form immigrant entrepreneurship intention. More specifically, we studied individual variables (previous entrepreneurial experience and perceived risk as a threat) and contextual variables (social network, entrepreneurial cultural distance, and institutional supports for entrepreneurship) as indirect antecedents, as well as attitudes, subjective norms, and perceived behavioural control as direct antecedents of IEI. We expected that individual and contextual variables would show indirect effects *via* attitudes, subjective norms, and perceived behavioural control. Direct impact on subjective norms was also being expected from attitudes and perceived behavioural control.

Regarding the direct antecedents of intention described by the personal attitudes, subjective norms, and perceived behavioural control, only ‘perceived behavioural control’ was not conclusive in predicting IEI. While this result contradicts previous literature which proposed that individuals’ decision to start their own business is influenced by the perceived feasibility judgment (self-efficacy: Boyd and Vozikis, 1994), it, however, corroborates subsequent studies by these same authors who see entrepreneurial self-efficacy as another cognition, also with intentional qualities, as derived from social learning theory (Bird, 2015). As an alternative to the direct effects of antecedents on entrepreneurial intention, only personal attitudes show an immediate effect on subjective norms, while the impact of ‘perceived behavioural control’ on subjective norms is not confirmed. This suggests that an immigrant’s self-efficacy does not seem to convince his or her reference group. In this study, the effect of the reference group’s social norms is difficult to identify due to the sample’s cultural diversity. Il can also imply that the need for starting an entrepreneurial venture for survival in a new country was so dire that immigrants entrepreneurs do not put that much important on the effect of subjective norms of their reference group. However, it cannot be excluded that we are dealing with some individuals with low self-efficacy beliefs: pessimistic self-efficacy beliefs are likely to affect behaviour, particularly thinking and decision-making quality (Bandura, 1997 cited by Reeve, 2012).

Pertaining to individual variables, the three psychological factors have helped to better understand the effect of these variables on immigrants’ intention to engage in entrepreneurship. They add an important perspective to the study on immigrant entrepreneurship by addressing why individual variables might be necessary for immigrants’ transition to entrepreneurship. In this vein, our study offers promising results on how individual variables intertwine as economic and psychological lenses to provide an overall picture, through an interdisciplinary approach that has often been suggested but rarely applied (Gartner, 2007) particularly among immigrants. This result confirms ‘experience’ as a source of self-efficacy, in line with Bandura’s work (Reeve, 2012). Moreover, as long as the perceived risk does not undermine the perceived feasibility of their business idea, immigrant entrepreneurs can create value for themselves with more or less risk in the host country. This risk mitigation is suggestive of their potential entrepreneurial resilience.

Regarding contextual variables, among the three TPB factors, only ‘perceived behavioural control’ was affected by direct effects of distance in entrepreneurial culture and institutional supports for entrepreneurship, and not by network support. As for cultural distance, any confusion among immigrants is likely to reduce perceived feasibility, which contributes to the feeling of ineffectiveness. Whereas for perceived support, it confirms verbal persuasion as a source of self-efficacy in line with Bandura’s work: the dynamic narratives of programs and other people’s experience tend to persuade the immigrant audience that they can competently carry out any entrepreneurial activity if they try (Reeve, 2012). The extra-ethnic social network of the immigrants appears not much helpful for their career plan. This emphasizes the lack of integration of immigrants in local networks. Although the reality of an ethnic enclave needs to be nuanced nowadays, this point nevertheless suggests that ethnic resources remain an asset. The strong ties of networks operating within the communities are a determinant factor for an ethnic business start-up serving the communities with the similar tastes and preferences.

In a nutshell, our results show that TPB partially accounts for overall indirect effects. Individual factors, indicators of experience and perceived risk all appear to operate within the broader framework of TPB in that they are linked to the IEI. On the other hand, the seemingly unsuccessful indirect effects of entrepreneurial culture, distance and institutional supports for entrepreneurship are probably due to the cognitive nature of both distance and support on feasibility. The result of (bridging) social networks, which seems counter-intuitive, rather calls for doing things differently (e.g., the need to develop ties with host country stakeholders to alleviate the relational risks of liability of foreignness imposed by local entrepreneurial culture on enterprising immigrants: horizontal social embeddedness achievement).

While attitudes and perceived behavioural control (PBC) are understood as the two strongest antecedents of immigrant entrepreneurship intentions (Ajzen, 1991), how can we understand the indirect effects of individual and contextual factors’ lack of transmission to the final intention? Two explanations seem plausible. First, the possibility of reverse causality has been raised in the previous literature. It has been suggested that an increase in IEI may affect desirability and feasibility (Brännback et al., 2007). Elfving et al. (2009) report that the idea of such reciprocal causality is reasonable. This suggests that the intention formation process would be iterative and therefore evolutionary (Krueger, 2017). Unfortunately, our sample did not allow us to test such a mechanism. Secondly, the result seems to challenge the performance of the entrepreneurial support of which some immigrants are beneficiaries. Discovering the gap between the entrepreneurial culture of their country of origin and that of the host country creates “a constant tension” (see Henríquez et al., 2021, p.2), a source of dissatisfaction (Hofstede et al., 2004) which looks like a double-edged sword. On the one hand, that dissatisfaction pushes them to start their business as individuals who do not conform to the predominant or mainstream direction of the country’s cultural dimensions (asset wise). At the same time, on the other hand, the tension created by cultural differences is likely to limit the effect of perceived behavioural control on intention (burden wise). Because when an important informal institution is different, the gap which undermines efficient and effective transactions of productive markets can be construed as informal institutional void (Webb et al., 2020). This tension can only be mitigated by institutional supports for entrepreneurship. This is the mission that entrepreneurial support structures should fulfill with immigrants in their entrepreneurial emergence process. Otherwise, the potential immigrant entrepreneur procrastinates, even has mental blockages and gives up in the face of obstacles. Therefore, the immigrants should use the opportunity of their mixed embeddedness in institutional context.

### 5.1. Theoretical implications

Theoretically, this study makes several important contributions to immigrant entrepreneurship. First, we connect immigration with entrepreneurship to integrate mixed embeddedness with the TPB, both conceptually and empirically on a cognitive level. Based on our assessment, both TPB and mixed embeddedness logics are important to understand the relevance of the IEI formation process. This indicates new theoretical linkages that have rich potential for theory and research in immigrant entrepreneurship. This study extends the TPB by contributing significantly to the limited literature on factors driving the immigrants’ intention process to become entrepreneurs in their host country. In this article, we are concerned with the antecedent drivers of IEI, and whether these are different from the drivers of traditional EI. Since both immigrant and traditional entrepreneurship are desirable for economic growth, global competitiveness and entrepreneurship diversity, it is important to understand how both types of entrepreneurships behaviour emerge. This empirical study confirms the plausibility of TPB in understanding the antecedents of intention to start a business by immigrants. It has clearly shown the importance of several factors that are often overlooked in previous works on IEI. The goodness of fit of the model validates the inclusion of these factors in the TPB when predicting EI of immigrants. Moreover, while the evidence from the literature shows that the TPB variables have been extensively studied in the decisions of individuals to engage with an intention to start a business in various contexts, studies that engage immigrant entrepreneurs’ intention formation process are rare. The inclusion of such previously unexplored factors fills important knowledge gaps and enriches the understanding of their effects on the EI of immigrants in host countries, under a holistic decision-making process (Douglas, 2020). Second, it also enriches the theory by comforting the TPB against “the problem of ‘inclined abstainers’, individuals who form an intention and subsequently fail to act, that has been a recognized limitation of the TPB” (Sniehotta et al., 2014, p.2). Third, it improves the understanding of the mixed embeddedness approach, by adding the effects of country of origin (which are part of cognition), a neglected aspect that recently drew criticism (Zhu et al., 2023). The approach is a Waldinger et al.’s (1990)  interactive model refined by adding new elements limited to the host country-specific institutional frameworks (Kloosterman and Rath, 2018). Finally, to the best of our knowledge, entrepreneurial culture distance in entrepreneurship research has not been adequately addressed previously. In doing so, this study advances theoretical conceptualization in the field of immigrant entrepreneurship. Furthermore, the IEI formation process cannot be adequately described without addressing the factors holistically, through learning and cultural sensitivity. This logical argument (close to cognitive embeddedness: Dequech, 2003) that underpins this study helped us to identify which factors should be studied, as well as how and why they are inter-linked.

### 5.2. Empirical implications

The operationalization of cultural distance of entrepreneurship concept is intended as an empirical contribution to existing knowledge on immigrant entrepreneurship. This study empirically tests an entrepreneurial model based on the relationship of belief-attitude-intention framework developed by several authors such as Krueger (2009). We present an integrative model integrating individual and contextual dimensions along with psychological factors that jointly form the immigrant entrepreneurship intention formation process. This study differs from the study of Zhu et al. (2023) where variables like entrepreneurial cultural differences were absent.

### 5.3. Practical implications

The importance of our research lies, first, in the fact it provides a valuable piece of information to those in charge of entrepreneurial support and decision-makers who seek to further promote the emergence of entrepreneurial activity in immigration context. Immigrants cannot claim to become entrepreneurs merely on the basis of an identity (Kirkley, 2016). As entrepreneurship is a learning process (Lin et al., 2023), even more so is entrepreneurial intention itself. We argue that EI is also a learning process with regards to the way enterprises are doing business. Second, analysis across the world’s entrepreneurial ecosystems displays common challenges posed by an entrepreneurial culture to develop and transmit and how to ensure that individuals who wish to start a business actually take action (GEM, 2020). The study affords clear implications of theory for problem-solving in immigrant integration situations. Immigrants should ‘learn to undertake’ for ensuring progress in the process. Rather than developing rigid policy instruments, our results allude to the importance of encouraging the entrepreneurial mindset and network of immigrants. The problem is not just taking action, but rather persisting in action. This requires integral embeddedness. We therefore support and plead for formal experience-based training. Third, if our results show that perceived risk can weaken the passion for entrepreneurship, entrepreneurial behaviour reality and contexts require individuals to get familiar with local entrepreneurial culture. Examining culture at a more granular level than previous research reveals that entrepreneurial culture is a shared liability issue: the liability of foreignness behoves the immigrant to learn the local entrepreneurial culture whereas the liability of the host country consists of cultivating such culture in a way that allows learning it to boost the entrepreneurial mindset. Empowering immigrants’ entrepreneurial intention through entrepreneurial culture is the key. Policy makers should take action to help their entrepreneurial ecosystems to thrive by strengthening human capital, addressing local/national inequalities through promoting cohesive, inclusive, and sustainable entrepreneurship. To support integration through entrepreneurship, destination countries should help the immigrants in improving their human and social capital. Consequently, this would lessen the disadvantage they face in their host countries. Finally, in international business literature, several recent studies have begun to focus on the joint effect of formal and informal institutions (Shen et al., 2023). The results of our study are consistent with this and call for institutional complementarity in the support to be offered to immigrant entrepreneurs.

## 6. Conclusion

Although it is not always easy to work at the intersection of two disciplines, this study could attest to the benefits of combining economic and psychological perspectives in examining IEI. It is challenging to appreciate how the cognitive perspective, in which we have embedded this work, has served as an interface to integrate several theoretical approaches. This has led to a deeper understanding of the process of IEI. The difficulties and obstacles being encountered by immigrants in starting and developing their businesses are often those that they should have paid attention to during the entrepreneurial intention phase. As the saying goes, “Trees with deep roots are those that grow high.” The challenges that are not identified at this stage can persist and jeopardize the career that is chosen out of opportunity or necessity. While perceived risk only affected intentions via the TPB’s desirability factors, the formal and informal context only had direct effects on the feasibility factor of the TPB framework. Particularly, future studies could focus more on a longitudinal analysis of these direct effects.

By and large, therefore, we conclude that the interaction between individual factors and contextual factors plays an important role in the transition of immigrants from the employability world to that of self-employment, and that future research should continue to shed light on this interaction, preferably in an interdisciplinary manner and especially, using longitudinal designs. This will contribute to a better understanding of immigrants’ EI for engaging in entrepreneurship. Moreover, the use of a structural equation model has confirmed that the distance between the host country’s entrepreneurial culture and that of the country of origin acts as a reducing factor in perceived feasibility. In contrast, entrepreneurial support should serve as an amplifier of the latter to maximize EI and, without further delay, move on to real entrepreneurial action. There would be a compromise in the perception of feasibility of a business project between entrepreneurial cognitive mechanisms, particularly between the distance of entrepreneurial culture and institutional entrepreneurial support. This study calls for an adaptation of the *ad hoc* structures to the actual support needs.

It should be noted that this study has several limitations. Although the hypothetical path model is based on well-established theories, unfortunately, the correlational design of our study does not allow for strictly causal interpretations. Another limitation is the fact that our data was collected from a single source: the immigrants. Canadian experience (human capital) of immigrants was only assessed using the single-item measure. While it is important to limit the number of items that respondents must complete, future research could also use multiple item instruments for this construct. Finally, IEI is a psychological state of mind and there are many more individual and social variables that could also be integrated into the model. We have also assumed Canada as a given background of space and place in relation to immigrant entrepreneurship (Webster and Kontkanen, 2021) except the entrepreneurial cultural difference, institutional supports, and personal network in the host market. Future studies on IEI might consider other individual, and contextual variables related to Canadian space and place that facilitate or create constraints in the IEI formation process. Future study might also consider variables such as the duration of stay in Canada and visa-type of immigrant entrepreneurs for the IEI formation process (Falcão et al., 2022).

## Data availability statement

The original contributions presented in the study are included in the article/supplementary material, further inquiries can be directed to the corresponding author/s.

## Ethics statement

The studies involving human participants were reviewed and approved by the Research Ethics Committee of Laval University. Written informed consent for participation was not required for this study in accordance with the national legislation and the institutional requirements.

## Author contributions

RM and ZS contributed to conception and design of the study. RM and CF oganized the database, performed the statistical analysis with MM. RM, ZS, MM, and CF wrote sections of the manuscript. All authors contributed to the article and approved the submitted version.

## Conflict of interest

The authors declare that the research was conducted in the absence of any commercial or financial relationships that could be construed as a potential conflict of interest.

## Publisher’s note

All claims expressed in this article are solely those of the authors and do not necessarily represent those of their affiliated organizations, or those of the publisher, the editors and the reviewers. Any product that may be evaluated in this article, or claim that may be made by its manufacturer, is not guaranteed or endorsed by the publisher.
